# Functional transition rate of the default mode network is associated with self-reported resilience

**DOI:** 10.1016/j.neuroimage.2025.121508

**Published:** 2025-10-06

**Authors:** Chun-Wei Hsu, Shulan Hsieh, Wan-Rue Lin, Ya-Ting Chang, Yu-Shiang Su, Cheng-Ta Yang, Yun-Hsuan Chang, Sheng-Hsiang Lin, Joshua Oon Soo Goh

**Affiliations:** a Graduate Institute of Brain and Mind Sciences, College of Medicine, National Taiwan University, Taipei 100, Taiwan; b Department of Psychology, National Cheng Kung University, Tainan 701, Taiwan; c Institute of Allied Health Sciences, National Cheng Kung University, Tainan 701, Taiwan; d Department of Public Health, National Cheng Kung University, Tainan 701, Taiwan; e Department of Education and Humanities in Medicine, Taipei Medical University, Taipei 110, Taiwan; f Institute of Gerontology, College of Medicine, National Cheng Kung University, Tainan 701, Taiwan; g Institute of Behavioral Medicine, College of Medicine, National Cheng Kung University, Tainan 701, Taiwan; h Institute of Clinical Medicine, College of Medicine, National Cheng Kung University, Tainan 701, Taiwan; i Biostatistics Consulting Center, National Cheng Kung University Hospital, College of Medicine, National Cheng Kung University, Tainan 701, Taiwan; j Department of Psychology, National Taiwan University, Taipei 100, Taiwan; k Neurobiology and Cognitive Science Center, National Taiwan University, Taipei 100, Taiwan; l Center for Artificial Intelligence and Advanced Robotics, National Taiwan University, Taipei 100, Taiwan

**Keywords:** Default mode network, Multi-voxel analysis, Psychological resilience, Functional state transitions, Neurocomputational dynamics, Mental state

## Abstract

Dynamic variations of information in our environment constantly influence our thoughts, requiring the brain to regulate its internal state transitions to maintain stable psychological functioning. Presumably, effective regulation of brain state transitions — defined as changes from one functional state to another over time — reflects psychological resilience whereas failure to adapt can lead to mental health challenges. However, the specific relationship between these dynamic functional changes and psychological resilience remains unclear. We evaluated neurocomputational changes of the default mode network (DMN) using indices of its functional transitions based on conventional regional mean responses as well as multi-voxel state dynamics, and examined their associations with self-reported resilience in a sample of 336 young adults (171 males, 165 females). Smaller multi-voxel DMN functional state transitions were specifically associated with greater perceived self-resilience, particularly in individuals reporting lower external support. Smaller transitions of DMN regional mean responses were positively associated with more generic resilience measures, though this appeared less robust and potentially susceptible to confounds such as head motion and the size of DMN regions. Associations between resilience and functional state transitions was specific to the DMN, with only limited contributions observed from sensory and salience networks. Our findings reflect a basis for making neurocomputational linkages between brain functional dynamics and subjective experiences. Potential applications for interventions are present for enhancing mental adaptability by modulating DMN transitions, offering a promising index for evaluating intervention outcomes and informing resilience-based mental health strategies.

## 1. Introduction

The variations of information we experience in the environment continually affect our thoughts. Amidst such dynamic external influences, the brain needs to regulate the transitions of its internal states in order to sustain psychological functioning (Buzsáki, 2019; Cieri et al., 2021; Friston, Breakspear, and Deco, 2012, 2010). An inability to engage appropriate neurocomputational strategies to balance environmental perturbations might result in mental health difficulties (Collins et al., 2011; Moitra et al., 2023). Nevertheless, how transitions between brain states are associated with psychological functioning remain relatively unexplored. Recent efforts have begun to characterize how large-scale brain networks dynamically reorganize over time, offering a promising framework to understand adaptive mental function (Bassett and Sporns, 2017; Lurie et al., 2020). In particular, the role of specific networks in these transitions may provide insights into neural mechanisms underlying psychological resilience.

We note that the default-mode network (DMN), which comprises the medial frontal, precuneus, and bilateral temporal-parietal regions, consistently dampens its mean activity when processing external events across a broad range of tasks (Buckner, Andrews-Hanna, and Schacter, 2008; see reviews in Fox and Raichle, 2007). Moreover, spontaneous neural activity fluctuations in the DMN have been linked to the integration of extrinsic and intrinsic information for a unified psychological state (Andrews-Hanna, Reidler, Huang, and Buckner, 2010a; Yeshurun, Nguyen, and Hasson, 2021). Given its role in internally directed cognition and self-relevant processing, the DMN may serve as a key neural substrate through which brain state transitions relate to resilience. Thus, it is possible that dynamic transitions in DMN functional activity have some indication for a person’s psychological stability amidst life experiences, i.e., resilience.

Psychological resilience in humans is generally described as the maintenance of normative mental function in the face of adversity (Masten, 2001). This is a complex construct involving different contextually inferred definitions of risks and assets influencing mental outcomes (Kaplan, 2002; Sisto et al., 2019). As such, it is difficult to experimentally measure psychological resilience. Nevertheless, in lieu of manipulating individual life situations, practical instruments are available in the form of subjective self-report questionnaires (Windle, Bennett, and Noyes, 2011). While these do not assess *in situ* behaviors in real-life challenges, they provide surrogates in indexing a person’s metacognitive perception of their ability to manage hypothetical adversity. These subjective ratings reflect self-evaluative judgement, which may be particularly linked to the DMN’s function in internal mentation. Thus, in this study, we sought to examine the associations between self-perceived resilience and DMN functioning. To this end, we used three self-report measures of psychological resilience that show high psychometric ratings (Windle et al., 2011): the Connor-Davidson Resilience Scale (CD-RISC) (Connor and Davidson, 2003), the Brief Resilience Scale (BRS) (Smith et al., 2008), and the Resilience Scale for Adults (RSA) (Friborg, Hjemdal, Rosenvinge, & Martinussen, 2003). The BRS is a validated tool that specifically measures an individual’s ability to “bounce back” from stress, while RSA and CD-RISC indices incorporate broader aspects of resilience, including external factors like family cohesion and social support. Crucially, to separate possible differential contributions of deeper facets of resilience metacognition, principal components derived from the above scales were comprehensively evaluated, and their relationships with DMN functional transitions then examined.

Previous research has found neural correlates of psychological resilience that implicate several networks, including the DMN (Iadipaolo et al., 2018; Long et al., 2019; Miyagi et al., 2020) and the salience network (Iadipaolo et al., 2018; Kong, Wang, Hu, and Liu, 2015), with some central executive network involvement (Iadipaolo et al., 2018). For instance, increased connectivity within the DMN (Shi, Sun, Wei, and Qiu, 2019) and regional homogeneity of low frequency fluctuations in the salience network (Kong et al., 2015) have been linked to resilience scores. Such findings identify a distributed neural basis for resilience that spans brain areas involved in self-related and task-related processing. As reviewed above, however, participants’ self-ratings on resilience scales (CD-RISC, BRS, and RSA) examined in this present study likely involve more metacognitive access into one’s general internal mental state that is not necessarily tied to immediate event salience or task execution processing. For instance, one can feel resilient in general, but exhibit non-resilient behavior when specific events happen; and vice versa, one can exhibit resilient behavior during an event, but report non-resilience in general. Therefore, in this study, we postulate resilience metacognition to reflect baseline internal mental state operations that are necessarily linked to the dynamic resting-state functional state changes of the DMN, given its established role in self-related processing. Whereas non-DMN functions might also relate to resilience as a complex construct, these are less focal in this study.

We considered that DMN regional mean response variances over time, measured in spontaneous resting-state functional magnetic resonance imaging (rsfMRI), reflects baseline regional neural activity dynamics. Smaller consecutive mean response changes index less metabolic fluctuation in the brain regions of interest, indicating less overall neural processing variation. More critically, we characterized the functional state of the DMN as the *multi-voxel* rsfMRI responses at a given moment, with a transition to another DMN state occuring when voxel responses change at the next moment. Such multi-voxel activation states can be projected into lower dimensional spaces using multidimensional scaling (MDS) (Cunningham and Yu, 2014; Friston, Frith, Fletcher, Liddle, and Frackowiak, 1996; Tzagarakis, Jerde, Lewis, Uğurbil, and Georgopoulos, 2009). The position differences between temporally consecutive projections can then indicate the velocity with which DMN functional states, reflecting each voxel’s neurocomputational contribution, changed during that period (see Methods). Importantly, smaller velocities index more autocorrelated consecutive DMN functional states. Note that previous analytical approaches to resting-state data (as in the above studies) used functional connectivity measures, such as time-series correlations between regions or voxel-wise coherence, to capture activity synchronization across spatially distributed areas, our method was specifically designed to capture dynamic changes in multi-voxel patterns over time. Following these definitions, there are two possible scenarios in this study. First, higher self-reported psychological resilience might reflect relatively stable DMN neurocomputational states, and as such should be associated with smaller DMN functional transitions. This hypothesis aligns with previous findings showing that lower brain network flexibility in the DMN region is associated with higher resilience (Long et al., 2019). Moreover, increased activity in DMN areas has been linked to maladaptive rumination, a hallmark of depressive symptoms and reduced resilience (Chou, Deckersbach, Dougherty, and Hooley, 2023; Zhou et al., 2020). Alternatively, higher self-reported psychological resilience might reflect greater modulation in DMN processing, and in this case should be associated with greater DMN functional transitions. This view is somewhat supported by evidence that stronger connectivity between DMN and basal ganglia was correlated instead with increased cognitive flexibility (Vatansever, Manktelow, Sahakian, Menon, and Stamatakis, 2016), a component of adaptive and resilient behavior. Thus, we examine our empirical data to more clearly determine which of these plausible alternatives hold.

In sum, we sought to determine the nature of the link between one’s resilience metacognition and the neurocomputational dynamics of one’s DMN. To do this, we developed, used, and evaluated the above novel methodology to calculate a DMN functional transition metric obtained from rsfMRI data and conducted a series of analyses to validate the above hypotheses of its associations with self-reported psychological resilience. Specifically, we evaluated multi-voxel DMN state transitions, as well as the root mean square of successive differences (rMSSD) and conventional standard deviations based on averaged activities of DMN regions-of-interest (ROIs). Further, we examined the artifactual effects of head motion and the number of voxels comprising individual DMN ROIs, and the specificity of DMN-derived functional transition indices versus non-gray matter regions. We expected the regional average time series index of DMN voxel responses to have associations with generic psychological processes (Huber et al., 2014), whereas its multi-voxels states should capture more specific neurocomputational aspects, each yielding distinct predictions for self-reported resilience. Functional transition associations in other non-DMN regions with resilience ratings were also examined, albeit these were of secondary interest.

## 2. Methods and materials

### 2.1. Participants

340 younger adults volunteered for this study. Exclusion criteria were the presence or history of neurological or psychiatric disorders, artificial implants, or other counterindications for MRI scanning. All participants underwent the MRI session followed by a self-report questionnaire session on a separate day within 11 months. Participants signed a written informed consent form approved by the Research Ethics Committee of National Cheng Kung University, Tainan, Taiwan, R.O.C. Four participants were excluded from data analysis due to excessive head movement during functional imaging (>3.4375 mm translation or >3° rotation). The remaining 336 participants (165 females) were between 20 and 34 years old (mean age = 22.68 yrs, SD = 2.85 yrs).

### 2.2. Resilience score measurement

The CD-RISC, BRS, and RSA resilience measures were selected based on (Windle et al., 2011), which applied established psychometric assessment criteria (Terwee et al., 2007). Our sample is based on Chinese-speaking Taiwanese participants such that we used validated Chinese versions of the measures. The Chinese version of CD-RISC (Connor & Davidson, 2003) consists of 25 items on self-perceived *ability to manage* general stress and adversity rated using 5-point Likert scales with total scores ranging from 0 to 100. The Chinese version of BRS (Smith et al., 2008) is a 6-item 5-point Likert-type assessment that indexes the *ability to recover* from stressful events, with the average total scores ranging from 1 to 5. The Chinese version of RSA (Friborg et al., 2006) is a 29-item 7-point Likert-type assessment that measures *intra-* and *inter-personal protective factors* with scores ranging from 29 to 203. Across these scales, higher scores indicate higher psychological resilience.

### 2.3. Brain imaging acquisition protocols

Brain imaging data were acquired on a GE MR750 3T scanner (GE Healthcare, Waukesha, W1, United States) with 32-channel head coils located at the Mind Research and Imaging Center and National Cheng Kung University (NCKU), Tainan, Taiwan. For each participant structural and functional images were acquired using the following parameters: A T1-weighted structural volume using a fast-spoiled gradient-recalled echo sequence consisting of 166 axial slices with scan time 218 s, repetition time (TR) 7.6 ms, echo time (TE) 3.3 ms, flip angle 12°, field of view (FOV) 22.4 × 22.4 cm^2^, slice thickness 1 mm, and matrix size 224 × 224; 245 resting-state functional volumes (eyes-open; fixation screen; first 5 vol discarded as dummies) using an interleaved T2*-weighted gradient-echo planar imaging sequence with TR 2 s, TE 30 ms, flip angle 77°, FOV 22 × 22 cm^2^, thirty-two 4 mm axial slices, voxel size 3.4375 × 3.4375 × 4 mm.

### 2.4. rsfMRI preprocessing

**Fig. 1.**
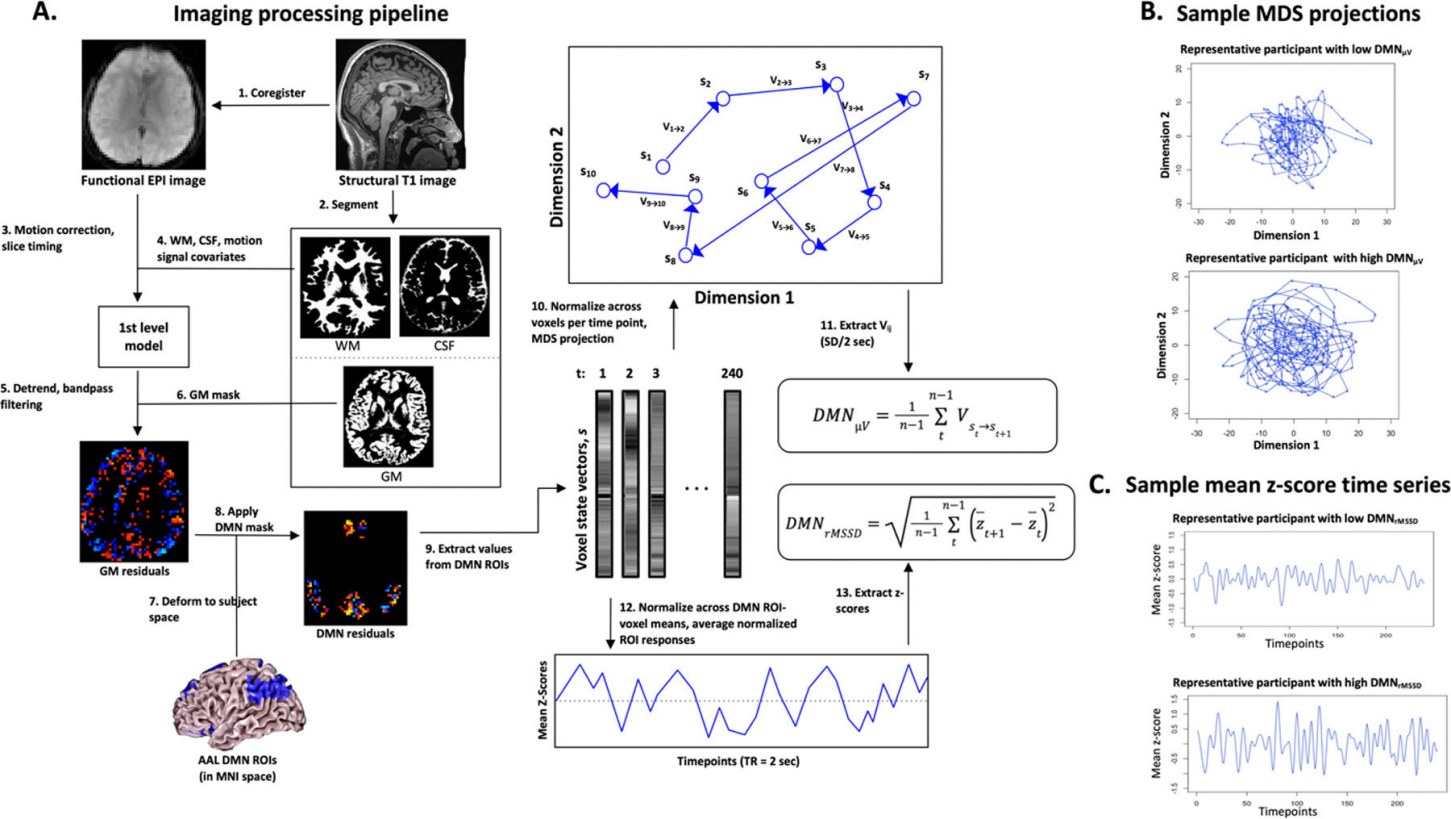
Image data processing pipeline for deriving individual participants estimates of DMN functional transitions DMN_μV_ and DMN_rMSSD_. **(A)** Schematic of the processing pipeline. **(B)** MDS projections from representative participants with low and high DMN_μV_. **(C)** Time series of DMN responses averaged across voxels from representative participants with low and high DMN_rMSSD_. DMN_μV_: Default-mode network mean state transition velocity; DMN_rMSSD_: Default-mode network root mean square of successive differences; EPI: Echo-planar image; WM: White matter; CSF: Cerebrospinal fluid; GM: Gray matter; AAL: Automated Anatomical Labeling; ROI: Regions of interest; MDS: Multi-dimensional scaling; TR: Repetition time; n: Number of time points.

Brain imaging data were preprocessed using SPM12 (Statistical Parametric Mapping, Wellcome Trust Centre for Neuroimaging, UK) (see Fig. 1). T1 structural images were first coregistered to functional images then submitted to the Diffeomorphic Anatomical Registration Through Exponentiated Linear Algebra procedure (DARTEL) to create a study-specific template (SST) (Ashburner, 2007) and individual deformation field maps. Functional images were submitted to head motion and slice-time correction. Non-neural noise was estimated using the anatomical component-based method (aCompCor) (Behzadi, Restom, Liau, and Liu, 2007) in the PhysIO toolbox (Kasper et al., 2017), yielding subject-specific time series estimates from WM and cerebrospinal fluid (CSF) masks and head motion covariates including frame displacements (FDs). These were regressed out from the preprocessed functional data. Resulting residual volumes were linearly detrended and bandpass filtered (0.08 to 0.009 Hz) with individual grey matter masks applied.

### 2.5. Default mode network (DMN) regions-of-interest (ROI)

Sixteen ROIs from the Automated Anatomical Labeling atlas 3 (AAL3) were used to define the DMN ROI (Rolls, Huang, Lin, Feng, and Joliot, 2020) (Table S1). These atlas-defined DMN ROIs were inversed-deformed into each participant’s native functional space using the corresponding individual deformation fields. Preprocessed filtered residual values from voxels in DMN ROIs inversed-deformed to subject-space were then extracted to derive functional transition indices (Fig. 1A; below). The number of voxels in participant inverse-deformed DMN ROIs, DMN_size_, were also obtained to examine its influence on DMN functional transition indices. To assess the robustness of our findings, we further replicated the analysis using the DMN parcels defined in the Schaefer 100-parcel functional atlas (Schaefer et al., 2018) based on the 7 and 17 network parcellation (Yeo et al., 2011).

### 2.6. Non-DMN ROIs

To assess the specificity of the observed DMN-resilience associations and possible effects in other non-DMN regions of secondary interest as well as control regions, we conducted parallel analyses using sensory and action networks and non-gray matter regions including ventricles (CSF) and white matter (WM). To define these ROIs, we categorized the AAL3 anatomical ROIs into ten functional sub-networks based on generally accepted functional attributions (see Table S1). Broadly, the sensory network ROI was then defined as including the primary sensory areas, secondary sensory areas, association (parietal, temporal) areas, medial temporal system, and limbic system. The action network ROI consisted of the monitoring system, executive system, premotor areas, and primary motor areas. For the ventricular ROI, we manually specified three geometric masks within the ventricles in the MNI template space using MarsBaR (Brett, Anton, Valabregue, & Poline, 2002). Specifically, the first was a box (with boundaries x: −5~5; y: 9~18; z: 3~12) spanning the right and left ventricles, the second was a sphere of 5 mm in radius in the third ventricle (centered at 0, −25, 12), and the third was a sphere of 5 mm in radius in the superior cistern (centered at 0, −40, −1). These were combined into a ventricular ROI which was then inverse-deformed to participant image space. As can be seen, these did not encompass the whole ventricle but were central regions within it. The reason we adopted this approach was to stringently avoid contamination of WM and GM signals for the multi-voxel functional state analyses, which we found was possible if using the CSF segmentation from preprocessing. Similarly, for WM, we manually specified three masks within the corpus callosum identified using the JHU-ICBM labels (https://identifiers.org/neurovault.collection:264). These were #4 genu of corpus callosum, #5 body of corpus callosum, and #6 splenium of corpus callosum; which were combined into a WM ROI inverse-deformed to participant image space.

Overall, the DMN was the primary network of interest for its established role in self-related processing and theorized link with resilience metacognition, the sensory and action networks were included as secondary comparative regions due to their potential involvement in resilience as in previous studies, and white matter and ventricular regions were control networks where we categorically do not expect associations between functional transition metrics and resilience ratings. In addition, we included saliency networks (i.e., Salience/Ventral Attention A and B) derived from the Schaefer atlas as further comparative networks based on other atlas definitions. As with the DMN ROIs, all ROIs, originally defined in MNI space, were normalized to the SST space and inverse-warped into each subject’s native space using individual deformation fields, thereby accounting for individual anatomical variability.

### 2.7. Multi-voxel DMN functional state transition indices

MDS was performed in R (version 4.0.2; R Foundation for Statistical Computing, http://www.R-project.org) using the “vegan” package. For each participant, multi-voxel data was extracted from the participant’s DMN voxels (in subject-space) to yield a state vector, s, consisting of the activities of all voxels at each time point, t=1, (Fig. 1A, Movie S1 & S2). There were 240 vectors comprising the resulting voxel × time point matrix per participant, in keeping with the number of functional volumes. Voxel-activity vectors for each time point, $S_{\tau }$, were normalized to the total participant matrix mean, i.e., z-transformed to SD units. $S_{\tau }$, were projected as state positions in lower dimensions using MDS with the projection stress value (a goodness-of-fit) evaluated to determine the appropriate dimensionality for each participant (Supplementary Materials). Transition velocities, $V_{s_{t}\to s_{t+1}}$, between states for consecutive time points, were computed as follows.


(1) $$\begin{array}{c} V_{s_{t}\to s_{t+1}}=\sqrt{\sum_{k}{\left(d_{t+1_{\left(k\right)}}-d_{t_{\left(k\right)}}\right)}^{2}} \end{array}$$

In Eq. (1) above, $d_{\left(k\right)}$ are the location coordinates of states, $S_{\tau },k$ represents 2, 3 or 4-dimensional space. In our fMRI data, the time between consecutive states is a constant TR=2s, so $V_{s_{t}\to s_{t+1}}$ Euclidean distance values are equated as having the velocity unit SD/2 s. Subsequently, mean functional state change velocities, ${DMN}_{\mu V}$, were defined as,

(2) $$\begin{array}{c} DMN_{\mu v}=\frac{1}{n-1}\sum_{t}^{n\;-\;1}V_{s_{t}\to s_{t+1}} \end{array}$$

where, n, is the number of time points per participant which is 240. Lower DMN_μV_ indicates slower mean state transition velocities, which we consider as reflecting higher functional *state* autocorrelation. i.e., all voxel activities comprising a state vector remain largely similar across temporally adjacent vectors. Action_μV_, Sensory_μV_, WM_μV_, and ventricles_μV_ from control ROIs were calculated similarly except that the respective ROI masks (Supplementary Materials, Table S1) were used to extract the voxel-activity state vectors.

### 2.8. Root mean square of successive differences in DMN ROI-mean functional transitions

For each participant, mean time series from the 16 DMN ROIs were extracted and normalized to the mean values of the ROIs for each time point. Normalized ROI time series were then further averaged to yield the mean z-scores of DMN responses for each time point (Fig. 1A). Successive differences were thus the differences between consecutive mean z-scores. DMN root mean square of successive differences (DMN_rMSSD_) was then calculated as,

(3) $$DMN_{rMSSD}=\sqrt{\frac{1}{n\;-\;1}\sum_{n-1}^{t}{\left({\overset{-}{z}}_{t+1}-{\overset{-}{z}}_{t}\right)}^{2}}$$

where, $\overset{-}{z}$, is the mean z-score of the ROI at time point τ. The key difference between DMN_rMSSD_ and DMN_SD_ is that the latter is based on differences of mean z-scores about the time series mean with no consideration of temporal order. Lower DMN_rMSSD_ and DMN_SD_ indicate smaller variations of mean ROI activity, which we consider as reflecting more similar general levels of resources recruited for neural processing in the brain regions of interest. Critically, we do not equate this with the functional state transitions indexed by DMN_μV_ as, conceptually, variations of means can be small even when the multi-voxel states have stochastic transitions.

## 3. Results

### 3.1. Principal components analysis (PCA) reveals general and intra-personal aspects of self-reported resilience

Unsurprisingly, significant positive correlations were found for CD-RISC with BRS (*r(334)* = 0.668, *p(FDR)* <0.001) and RSA (*r(334)* = 0.709, *p(FDR)* <0.001), and for BRS with RSA (*r(334)* = 0.534, *p(FDR)* <0.001). Indeed, PC1 positively loaded on all three scales consistent with a general index of perceived current ability to be resilient (Table 1). Importantly, PC2 had a positive loading on BRS, a negative loading on RSA, and minimal contribution from CD-RISC.

To elucidate the sign differences in PC2 loadings for BRS and RSA, we noted that the RSA consists of subscales that can be dissociated into intra- and inter-personal factors, whereas the BRS measures a more uniform construct (see Methods). Therefore, we performed a second PCA using the five RSA subscales (i.e., family cohesion, social competence, social resources, personal strength, and future structured style) along with the CD-RISC and BRS (see Table S2; subscript 2 denotes PCs from the secondary PCA). Like the main PCA analysis, PC1_2_ positively loaded on all the measures and subscales, indicating a general index of perceived current ability to be resilient. Critically, PC2_2_ had positive loadings on family cohesion, social competence and social resource subscales of the RSA, and negative loadings on the BRS, CD-RISC, and personal strength and future structured style subscales of the RSA. As expected, the negative loadings applied to subscales to do with intra-personal factors whereas the positive loadings applied to subscales related to interpersonal support (Friborg et al., 2003), which comprise nearly two-thirds of the RSA scale. As such, it is likely that mean RSA scores predominantly represented self-reported resilience based on having interpersonal support.

Thus, we further validated that the negative loading of PC2 on mean RSA scores reflects individual ratings on inter-personal subscales of the RSA, which is distinct from the intra-personal subscales. We defined a composite difference score [1/2*(Personalstrength+Futurestructuredstyle)-1/3*(Familycohesion+Socialcompetence+Socialresources)], which characterizes the degree to which a person expresses greater intra-than inter-personal factors in the RSA. This composite score was positively correlated with PC2 (*r(334)* = 0.231, *p* < .001), as expected. Taken together, these findings suggest that PC2 selectively indexes the degree to which individuals perceive themselves as able to recover from challenges as captured in the BRS, amidst minimal availability of interpersonal support, as reflected in the RSA. With this determination, PC1 and PC2 were then evaluated for their relationships with DMN functional transition indices.

### 3.2. Smaller DMN functional transitions were associated with higher resilience

**Fig. 2.**
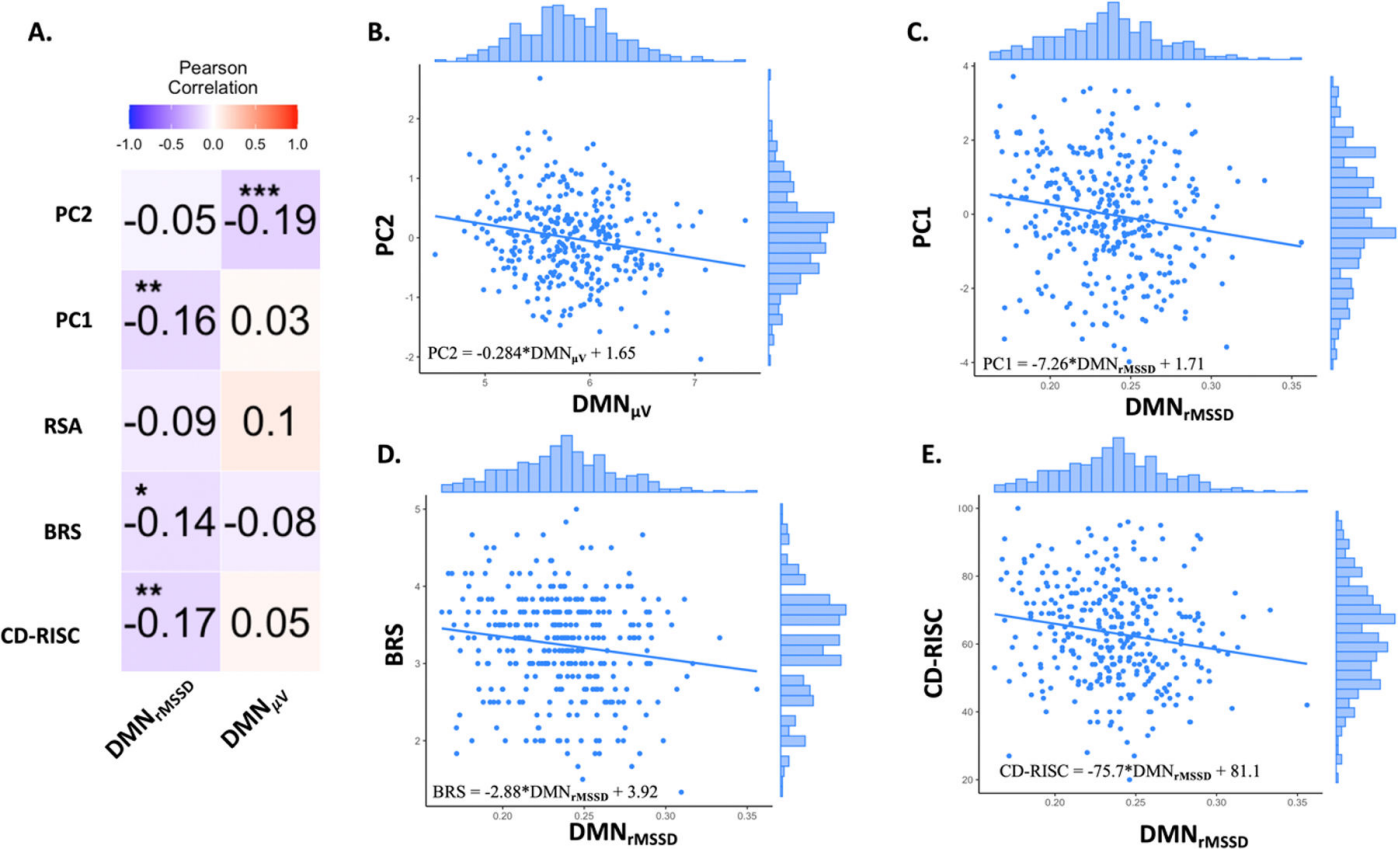
Pearson’s correlations between DMN functional transition indices (DMN_μV_ and DMN_rMSSD_) and psychological resilience measures, and PCs 1 and 2. **(A)** Heatmap for Pearson’s correlations. **(B) to (F)** Scatterplots of significant correlations with distribution histograms on the axes. *, ** denote p(FDR) < 0.05, 0.01, respectively. Equations denote regression functions of the effect of the DMN indices on the resilience measures (trend lines in plots). FDR: False Discovery Rate; CD-RISC: Connor-Davidson Resilience Scale; BRS: Brief Resilience Scale; RSA: Resilience Scale for Adults; PC1, PC2: Principal Components 1 and 2 (see Table 1); DMN_μV_: Mean default-mode network multi-voxel state transition velocity; DMN_rMSSD_: Root mean square of successive differences in default-mode network (time series averaged across voxels).

Pearson’s correlation analyses were conducted to examine the relationships between DMN functional transition indices and the psychological resilience PCs. Critically, DMN_μV_ had a significant negative correlation with PC2 (*r(334)* = −0.193, *p(FDR)* <0.001) (Fig. 2A, and B; see Supplementary Materials for replication and analysis using Schaefer atlas). By contrast, DMN_rMSSD_ had significant negative correlations with CD-RISC (*r(334)* = −0.172, *p(FDR)* = 0.003), BRS (*r(334)* = −0.143, *p (FDR)* = 0.014), and PC1 (*r(334)* = −0.157, *p(FDR)* = 0.007)(Fig. 2A, D, E, and F). However, these associations were weaker in the first sub-batch (Supplementary Materials, Fig. S2). Thus, individuals with smaller DMN functional response transitions perceived themselves as more resilient. Moreover, DMN_μV_ was specifically associated with PC2 whereas DMN_rMSSD_ implicated more generic resilience.

Note that across participants, the basic effect sizes of DMN_μV_ had a mean (SD) of 5.80 (0.464) SD/2 s and DMN_rMSSD_ had a mean (SD) of 0.236 (0.033) SD/2 s, and as expected, the two positively correlated at *r (334)* = 0.194, *p(FDR)* <0.001. Thus, it is interesting to appreciate that the DMN_μV_ is approximately 5 times larger than DMN_rMSSD_, with 38.8 % (0.194^2^) common variances. These descriptives, reported for interested readers and future reference, quantify the different base rates at which brain functional information changes when using either metric and the degree to which they capture unique aspects of neural activity in the same functional data.

### 3.3. DMN state transitions and possible confounds

To evaluate the robustness of the observed associations between DMN functional transitions and resilience, we examined potential confounding factors, including head motion and the size of DMN regions. Although brain image motion correction was applied during preprocessing (see Methods), residual head movement could still influence DMN state transitions indices. We found that mean framewise displacement (FD_mean_) did not correlate with DMN_μV_ (*r(334)* = −0.086, *p(FDR)* = 0.115) although it significantly correlated with DMN_rMSSD_ (*r (334)* = 0.149, *p(FDR)* = 0.009). Nevertheless, considering FD_mean_ as a covariate did not alter the correlation effect between DMN state transitions and resilience scores (Supplementary Materials), suggesting that the main findings were not driven by head motion.

We also considered whether individual differences in the size of DMN regions (DMN_size_) might influence DMN transition indices. We found that DMN_size_ (Methods) did not correlate with DMN_μV_ (*r(334)* = −0.091, *p(FDR)* = 0.097) although it significantly correlated with DMN_rMSSD_ (*r(334)* = −0.183, *p(FDR)* = 0.001). Again, including DMN_size_ as a covariate did not alter key correlation effects between DMN state transitions and resilience scores (Supplementary Materials), indicating that these results were not confounded by differences in DMN region size. Together, these analyses suggest that DMN_μV_ is relatively robust against potential confounds such as head motion and DMN region size, whereas DMN_rMSSD_ appears to be more susceptible to these influences.

### 3.4. Resilience measures and multi-voxel state transitions in non-DMN ROIs

**Fig. 3.**
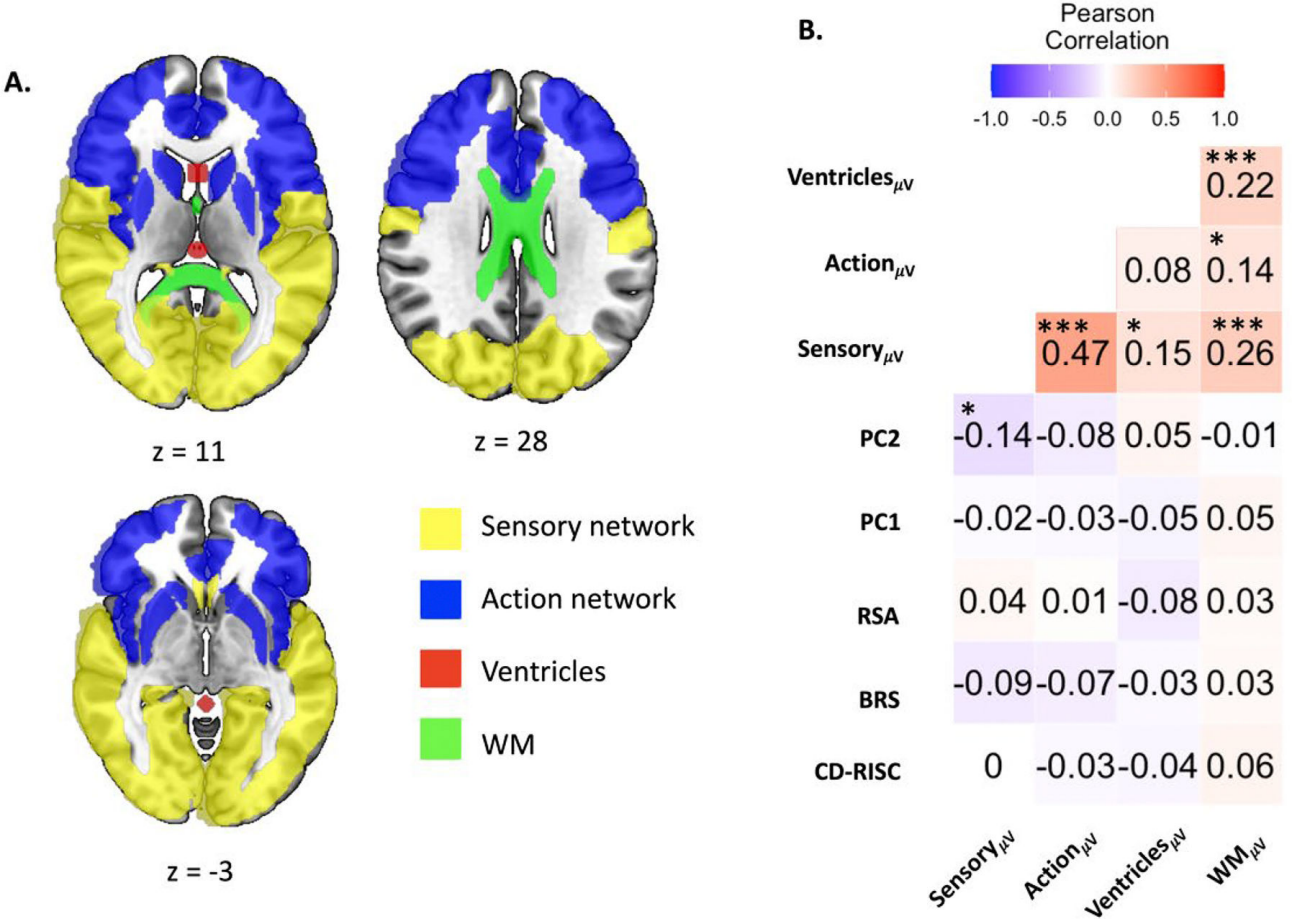
Axial slices depicting control ROIs in the brain in MNI template space and Pearson’s cross-correlations between mean state transition velocities in control ROIs, psychological resilience measures, and PCs 1 and 2. **(A)** 3 axial slices illustrating control ROIs (*z* = −3, 11, 28). **(B)** Heatmap representing Pearson’s cross correlations.*, **, and *** denote *p(FDR)* < 0.05, 0.01, and 0.001, respectively. MNI: Montreal Neurological Institute; ROI: Regions of interest; FDR: False Discovery Rate; CD-RISC: Connor-Davidson Resilience Scale; BRS: Brief Resilience Scale; RSA: Resilience Scale for Adults; PC1, PC2: Principal Components 1 and 2 (see Table 1); Sensory_μV_, Action_μV_, Ventricles_μV_, WM_μV_: Mean multi-voxel state transition velocities for sensory network, action network, ventricles mask, and white-matter mask, respectively.

To determine contributions of secondary non-DMN ROIs to PC2, and to validate the specificity of the above correlation between DMN_μV_ and PC2, we conducted parallel analyses using multi-voxel state transition indices derived from sensory and action networks, and also control ROIs in the ventricles (CSF) and white matter (WM) (see 2.5, Table S1). Among these ROIs, only Sensory_μV_ showed a significant negative correlation with PC2 (*r(334)* = −0.135, *p(FDR)* = 0.034) with no other regions showed significant correlations with any resilience measures (Fig. 3). Critically, functional transition indices in control CSF and WM ROIs were not associated with resilience ratings, as expected. The negative association between the sensory network and resilience was unexpected. It is possible that smaller functional state transitions in brain areas implicated in perceptual representations might also be indicative of an individual’s sense of self-resilience. In addition, additional analyses of the saliency networks based on the Schaefer atlas also revealed negative correlations with PC2 (Supplementary Information, Fig. S5). Interestingly, as seen in the supplementary analyses, the associations between functional state transitions in the Saliency networks with resilience ratings were dependent on voxels that overlapped with our DMN ROI definition. Together, these results support the primary DMN involvement in resilience with possible contribution from salience or sensory regions.

### 3.5. Conventional DMN time series standard deviation similar to dmn_rMSSD_

To evaluate whether traditional metrics of temporal variability show similar associations with resilience, we analyzed the standard deviation of DMN time series responses (DMN_SD_), calculated from non-consecutive, averaged and normalized responses across ROIs. DMN_SD_ significantly correlated with DMN_rMSSD_ (*r(334)* = 0.833, *p*(FDR) < 0.001) but not DMN_μV_ (*r(334)* = −0.017, *p*(FDR) = 0.757). Similar to DMN_rMSSD_, there were significant negative correlations between DMN_SD_ with PC1 (*r(334)* = −0.186, *p*(FDR) = 0.001) and the resilience measures (CD-RISC: *r(334)* = −0.201, *p*(FDR) < 0.001; BRS: *r(334)* = −0.146, *p*(FDR) = 0.010; RSA: *r(334)* = −0.130, *p*(FDR) = 0.022) but not with PC2 (*r(334)* = −0.013, *p*(FDR) = 0.869) (see replication in Supplementary Materials). DMN_SD_ had a significant positive correlation with FD_mean_ (*r(334)* = 0.120, *p*(FDR) = 0.041) and a significant negative correlation with DMN_size_ (*r(334)* = −0.135, *p*(FDR) = 0.039). Overall, these results indicate that DMN_SD_, the more conventional measure of DMN signal modulation, is susceptible to potential confounds such as head motion and DMN region size, similar to DMN_rMSSD_.

## 4. Discussion

The brain is a complex neural network in which dynamic activities enact computations that give rise to mental state variations (Cunningham and Yu, 2014). Thus, in fMRI data, it is important to consider the unique contribution of every voxel’s responses and their transitions from prior responses. In support, smaller transition rates in DMN multi-voxel states were selectively associated with a greater sense of psychological self-resilience across individuals. Conventional approaches using averaged DMN regional response transition sizes also showed generic negative associations with resilience measures, albeit these were less robust. Our findings motivate evaluations of functional brain state transitions as a voxel-level information metric of dynamic neural computations leading to specific psychological experiences.

### 4.1. DMN functional transitions and psychological resilience

Resilience as measured using extant behavioral scales encompasses not only the psychological abilities of the self but also external contexts, such as social and economic support, that bear on an individual’s perceived ability to manage life’s situations (Luthar, Doernberger, and Zigler, 1993; Masten, 2001; Windle et al., 2011). Here, we note that DMN_μV_ was not associated with generic indicators of resilience such as PC1. Rather, DMN_μV_ predicted psychological resilience specifically for PC2, which captured an individual’s perceived self-capacity to recover from adverse events amidst lower levels of external support. Such de-contextualized meta-cognition of self-functioning aligns with our hypothesis of a deep neural mechanism in the DMN that regulates psychological states amidst external circumstances. We speculate that smaller DMN multi-voxel transitions reflect neurocomputational mechanisms that integrate various incoming external experiences in a more controlled manner to sustain consecutively similar default states (Andrews-Hanna et al., 2010b; Buckner et al., 2008, 2009; Buckner and DiNicola, 2019). Overall, we demonstrate detectable associations between dynamic multi-voxel state transitions and self-reported mental states and provide insight into neural mechanisms underlying psychological stability and resilience.

DMN transition indices based on regional-averaged data, DMN_rMSSD_ and DMN_SD_, showed similar associations between brain functional variations and generic resilience measure ratings. These functional measures were correlated with head motion, granted that associations with resilience measures remained after adjusting for them as covariates. Nevertheless, the influence of motion artifacts in mean ROI signals cannot be easily discounted. Moreover, analytically, averaging responses across voxels strengthens common signals, including local broader vascular or metabolic states, and reduces contributions from signals unique to specific voxels, which are regarded as idiosyncratic. This reduces potentially informative voxel-level neurocomputational data. Our findings suggest that, to examine dynamic brain states, contributions of individual voxels should be regarded as complementary to ROI-mean approaches.

### 4.2. Multi-voxel state transitions and criticality in the DMN

Our evaluation of multi-voxel state transitions in the DMN follows other studies using reduced dimensions of multi-unit neural data to characterize brain states (Bassett and Sporns, 2017; Cunningham and Yu, 2014). For instance, microelectrode array recordings of monkey V4 neurons projected on a reduced attentional state dimension predicted eye movement behaviors (Cohen and Maunsell, 2010). In zebrafish, whole-brain calcium imaging of individual neuronal activities projected onto reduced dimensions revealed functional states corresponding to locomotive interactions with the visual environment (Ahrens et al., 2012). Moreover, differential flux in state transition probabilities in human whole-brain fMRI data captured different degrees of temporal non-reversibility of brain states during tasks and at rest, indexing non-equilibrium processing (Lynn, Cornblath, Papadopoulos, Bertolero, and Bassett, 2021). Here, we found support for multi-voxel state transitions predicting individual mental states selectively. Moreover, the dimension reduction approach affords expression of brain state changes in terms of velocity of normalized transitions, SD/*sec*. Such quantification of change rates of brain activity measured using fMRI might be explored in subsequent studies for linkages with the information processing capacity (e.g. bit rate) of neural systems.

The brain has been described as operating at an optimal level of information processing, termed criticality, which involves neural network activities that fluctuate within a narrow range of ordered and disordered states (Beggs, 2022; Chialvo, 2010; Cocchi, Gollo, Zalesky, and Breakspear, 2017). This is contrasted with the alternatives of completely fixed or random states of neural network activities. Criticality in neural networks has several information processing advantages including farther transmission ranges, greater storage capacity, greater range of operations (computational complexity and power), and stability of operations (Beggs, 2008). These properties might be considered for characterizing DMN operation in its role as a dynamic balancing network that has to adaptively negotiate between various internal neural processes and external environmental demands (Buckner et al., 2008). DMN functioning that is closer to criticality can afford better repertoires of computations and more extensive control to coordinate mental processes in the whole brain and thereby sustain psychological resilience as opposed to settling on non-adaptive mental states.

In our findings, individuals with lower DMN state transition velocities might exhibit criticality in that multi-voxel response patterns have minimal, but not zero, state fluctuations between consecutive moments. By contrast, individuals showing DMN states with large transitions might indicate greater stochasticity in multi-voxel fluctuations and are further from information processing criticality. It is intriguing to also consider the use of such information metrics to account for brain functioning in clinical cases like Alzheimer’s disease (Niu et al., 2018; Wang et al., 2017; Wang and Alzheimer’s Disease Neuroimaging Initiative, 2020). Thus, future studies might further pursue validating DMN transitional states as a biomarker of neurological or psychiatric pathologies, or as an intervention outcome indexing enhanced information-processing for navigating the complexities of life.

### 4.3. Limitations and future studies

It is well noted that the effect size observed for the key correlation result between DMN_μV_ and PC2 was *r* = −0.19 is relatively small, albeit significant under multiple comparison corrections. We highlight that small effect sizes are not uncommon in the context of complex human brain processes and behavior (Gratton, Nelson, and Gordon, 2022). A recent study (Marek et al., 2022) correlated brain and behavioral measures across thousands of individuals from three large consortia datasets and found a maximum |r| = 0.16 and mean |r| = 0.01 despite well-powered approaches. Given our sample size is not in the thousands, our findings should be viewed as initial evidence supporting an association between DMN functional transition metrics and self-reported resilience. Nonetheless, we supply analyses to evaluate the reliability and validity of our findings, with key associations generally replicated across sub-batches in this study (Supplementary Materials). Future investigations with larger and more diverse cohorts will be critical for evaluating the generalizability and practical relevance of these brain-resilience associations. Examinations of the effects of age, culture, or ethnicity might also reveal modulations of associations between DMN transitional rates and psychological states across different sample contexts.

To validate a fMRI-based metric against established resilience measures, it was necessary here to examine associations between objective biological measures with subjective behavioral ratings on resilience questionnaires. However, we recognize that self-report questionnaires may not accurately reflect real-life responses to adversity or a person’s actual capacity to recover, as they reflect more metacognitive perception of how individuals think they would react. As such, to derive more objective metrics of psychological resilience, it is necessary to circumvent potential biases in instruments based on subjective self-reports of perceived resilience ability. We suggest that there is a need for continued validation of brain functional transitional rates as an objective quantification of mental states with further examinations of associations with other biological factors (e.g., genetics, blood-based indicators) or performances in standardized tests of cognitive ability. Additionally, longitudinal data is needed to track the trajectory of mental health over time and further refine these assessments. Moreover, apart from examining spontaneous brain activity during rest, changes in functional transitional rates might be assessed under varying task goals, offering greater insight into how DMN state dynamics are influenced by immediate environmental challenges and how they are reinstated post-perturbation.

Beyond its classical characterization as a task-negative network, the DMN is increasingly understood as an active system involved in a wide range of cognitive functions (Menon, V., 2023; Raichle, M.E., 2015), including the integration of perceptible events and internally direct processes (Chiou, Humphreys, and Lambon Ralph, 2020; Zhang et al., 2022). These functional properties suggest that the DMN’s role in psychological resilience may be more nuanced than previously assumed. While the current study examined dynamic properties of the DMN as a unified network, future research could more deeply investigate the temporal dynamics of DMN subregions to better characterize their specific contributions to psychological resilience. Additionally, although we used atlas-defined DMN ROIs warped into each participant’s native space, DMN ROIs based on subject-specific coordinates such as those derived from resting functional data using independent components analysis (ICA) could provide finer individual-level precision. However, these approaches might first require more specialized designs beyond resting-state fMRI, such as multiple sessions or task-modulations, to help validation of the functionally derived DMN ROIs.

Interestingly, the observed negative correlations between our sensory and Schaefer et al.’s salience network state transitions and PC2 underscores the possibility that resilience is shaped not only by internal self-referential processes but also by how these processes interface with ongoing perceptual input and attention monitoring to potentially impact an individual’s metacognition. Indeed, given that salience networks are involved in detecting and filtering relevant stimuli for internal processing, this result suggests that more stable dynamics in these networks may also support the maintenance of coherent self-referential processing under varying internal and external demands. Also, the sensory network itself, particularly the visual cortex (Bergmann et al., 2024), is known to be involved in internal mental imagery independent of external input (Pearson, 2019). Mental imagery is closely linked to metacognition (Pearson, Rademaker, and Tong, 2011), since the ability to monitor and control internal mental representations is critical for evaluating the accuracy and reliability of one’s thoughts and perceptions. These non-DMN findings align with emerging evidence that the DMN is not a functionally uniform system, but instead comprises heterogeneous subsystems, some that interface with perceptual and semantic information or attention control, while others remain functionally decoupled from external input (Zhang et al., 2022). Future experimental research is needed to further disentangle the sub-roles of internal states (in DMN) and perceptual/attention processing (in sensory/salience networks) in shaping metacognitive aspects of resilience. In addition, more detailed evaluations across different network templates, e.g., the 7- and 17-network parcellations proposed by (Yeo et al., 2011), would allow for a finer-grained understanding of the functional subdivisions and their contributions to resilience, and could serve as a valuable direction for replication and extension in future studies.

### 4.4. Conclusion

In conclusion, both multi-voxel and ROI-mean indices show that lower DMN transition rates are associated with greater psychological resilience, although each captured different dimensions of resilience. Multi-voxel state transitions were more sensitive to perceived personal competence amid reduced external support availability, while regional-averaged data reflected a broader, more generalized aspect of resilience. Mental health issues facing humans have grown in recent years (Moitra et al., 2023) and maladaptive psychological responses to societal stressors (i.e., social discrimination, economic insecurities) and social media or general information exposure are key determinants of mental health disorders. In this light, this present initial work relating self-ratings of psychological resilience to an objective metric of variability in DMN functional states is a simple but important step towards a neurocomputational linkage between phenomenological experiences and brain function. Our findings offer a framework for formally describing neural mechanisms supporting similar consecutive brain states under normative conditions — and how they falter under stress or adversity. Consequently, formal models can be developed to predict conditions that modulate brain transitional rates and accelerate the designing of interventions and preventative strategies to fortify neural operations against non-resilience to adverse environments.

## Supplementary Material

Supplementary Material

MovieS1

MovieS2

Supplementary material associated with this article can be found, in the online version, at doi:10.1016/j.neuroimage.2025.121508.

## Figures and Tables

**Table 1:** Factor loadings of the PCs from principal component analysis of the resilience measures (CD-RISC, BRS, and RSA) with percentages of total variance accounted for in parentheses and the means of the measures.

	Mean (SD)	PC1 (75.9 %)	PC2 (15.6 %)	PC3 (8.51 %)

CD-RISC	63.3 (14.4)	0.915	−0.055	−0.400
BRS	3.24 (0.66)	0.838	0.517	0.176
RSA	144 (23.1)	0.858	−0.446	0.254

SD: Standard Deviation; PC: Principal Components; CD-RISC: Connor-Davidson Resilience Scale; BRS: Brief Resilience Scale; RSA: Resilience Scale for Adults.

## Data Availability

Data will be made available on request.

## Data and code availability statement

As part of our commitment to transparency and reproducibility, we fully support data sharing practices. Example dataset and the code used for analysis of mean velocities have been deposited at figshare and is publicly available at https://doi.org/10.6084/m9.figshare.27998954.v1.

However, we have opted to share our brain imaging data through direct requests rather than depositing it in an online repository. This decision was made to ensure that any requests for data are handled with appropriate consideration and oversight.
