# Supplementary material for: Functional transition rate of the default mode network is associated with self-reported resilience
Source: Neuroimage. Author manuscript; Available in PMC 2026 Jun 2. (PMC13229582; doi:10.1016/j.neuroimage.2025.121508)
Supplement: Supplementary Material [file NIHMS2170243-supplement-Supplementary_Material.docx]

**Supplementary Material**

*1. Projection stress scree plot evaluation*

    To determine the optimal MDS dimensionality for projecting multi-voxel DMN functional states, we evaluated dimensionalities ranging from 2 to 10 and calculated the average stress value at each dimensional size across all 336 subjects. The results are visualized in the Scree plot in Fig. S1, which shows a reduced group-level change in stress values between using 4 to 5 dimensions than between using 3 to 4 dimensions. This suggests that MDS dimensionality should generally not exceed 4 in our sample. To achieve a balance between model fit and complexity, we adopted a flexible dimension approach and applied a stress value threshold of < .15 as a criterion to balance the model complexity and fit quality for adequacy per participant’s projection dimensions [(Borg & Groenen, 2003)](https://sciwheel.com/work/citation?ids=6736572&pre=&suf=&sa=0). Consistent with the group-level Scree plot, all participants’ dimensional sizes were < 5 using this criterion. Accordingly, multi-voxel data for 20 subjects were represented in 2-D space, 296 subjects in 3-D space, and 20 subjects in 4-D space. These dimensions were chosen to minimize distortion while preserving the relative Euclidean distances between data points in the reduced space.

*2. Examination of replication across sub-batches in the grand study*

    The 336 participants included in the main analyses were effectively collected in two large batches (batch 1: n = 189, batch 2: n = 147) within approximately 4 years due to funding cycles and project implementation timelines. The first batch consisted of 189 participants (males/female = 92/97; mean age (SD) = 21.8 (1.96) yrs, age range 20 to 30 yrs) and contributed individual scores on the CD-RISC, BRS, and RSA with means (SDs) 65.14 (14.21), 3.26 (0.61), and 146 (22.5), respectively. Similar to the main results, significant positive correlations were found between CD-RISC with BRS (*r* = 0.651, *p(FDR)* <.001) and RSA (*r* = 0.701, *p(FDR)* <.001), and between BRS with RSA (*r* = 0.529, *p(FDR)* <.001). PCA showed that PC1, PC2, and PC3 for this sub-batch data explained 75.2%, 15.8%, and 8.96% of the total variance, respectively. PC1 positively loaded on all three scales (CD-RISC: *r* = 0.910, *p* <.001; BRS: *r* = 0.832, *p* <.001; RSA: *r* = 0.858, *p* <.001) consistent with a general index of resilience common across the measures. PC2 had a positive loading (0.528) on BRS, a negative loading (-0.437) on RSA, and minimal contribution from CD-RISC (-0.070).

    These behavioral results were also similar when examining the second batch of 147 participants separately. Significant positive correlations were found between CD-RISC with BRS (*r* = 0.695, *p(FDR)* <.001) and RSA (*r* = 0.707, *p(FDR)* <.001), and between BRS with RSA (*r* = 0.541, *p(FDR)* <.001). PCA showed that PC1, PC2, and PC3 for this sub-batch data explained 76.6%, 15.3%, and 8.07% of the total variance, respectively. PC1 positively loaded on all three scales (CD-RISC: *r* = 0.920, *p* <.001; BRS: *r* = 0.849, *p* <.001; RSA: *r* = 0.855, *p* <.001) consistent with a general index of resilience common across the measures. PC2 had a positive loading (0.489) on BRS, a negative loading (-0.469) on RSA, and minimal contribution from CD-RISC (-0.02).

    For the first batch, DMN_μV_ had a mean (SD) of 5.84 (0.455) SD/2 sec and DMN_rMSSD_ had a mean (SD) of 0.236 (0.032) SD/2 sec, with the two positively correlated at *r* = 0.226 (*p(FDR)* =.004). DMN_μV_ had a significant negative correlation with PC2 (*r* = -0.209, *p(FDR)* = .008, Fig. S2) and BRS (*r* = -0.208, *p(FDR)* = .008, Fig. S2). In addition, DMN_rMSSD_ had a significant negative correlation with CD-RISC (*r(334)* = -0.167, *p(FDR)* = .038, Fig. S2). DMN_SD_ correlated with DMN_rMSSD_ (*r* = 0.821, *p(FDR)* < .001) but not with DMN_μV_ (*r* = -0.012, *p(FDR)* = .872). DMN_SD_ significantly negatively correlated with PC1 (*r* = -0.208, *p(FDR)* = .006), CD-RISC (*r* = -0.232, *p(FDR)* = .002), and RSA (*r* = -0.164, *p(FDR)* = .033) but did not correlate with PC2 (*r* = 0.035, *p(FDR)* = .633), or BRS ((*r* = -0.141, *p(FDR)* = .066).

For the second batch, the correlation between DMN_μV_ and PC2 did not pass corrected significance but still showed a consistent uncorrected marginal negative trend (*r* = -0.160, *p(uncorrected)* = .053) (Fig. S3). In addition, there was a significant positive correlation between DMN_μV_ and RSA. This positive association suggests that a sense of resilience stemming from inter-personal factors might indicate reduced intra-personal self-resilience indexed in DMN functional transitions. Consistent with a more generic index, DMN_rMSSD_ showed negative correlations with PC1, BRS, and CD-RISC, although these associations did not reach statistical significance (Fig. S3). DMN_SD_ also showed negative correlations with PC1 (*r* = -0.157, *p(FDR)* = .086), CD-RISC (*r* = -0.174, *p(FDR)* = .059) and BRS (*r* = -0.149, *p(FDR)* = .099), although these associations did not reach statistical significance.

*3. Validation of DMN transition rates using Schaefer brain functional parcellation*

    To assess whether commonly used parcellations in the literature still demonstrated the negative relationship between DMN transition rates and resilience measures, we utilized the 7- and 17- network parcellations by Schaefer et al. (2018) [(Schaefer et al., 2018)](https://sciwheel.com/work/citation?ids=4939207&pre=&suf=&sa=0) to define DMN regions and calculate the velocity of DMN functional states (Fig. S4). The mean (SD) DMN_μV_ for the Schaefer 7 networks was 5.82 (0.468) SD/2 sec and for the Schaefer 17 networks, it was 5.85 (0.485) SD/2 sec, with a strong positive correlation between the two (*r* = 0.889, *p(FDR)* <.001). PC2 showed a significant negative correlation with DMN_μV_ from both the Schaefer 7 (*r* = -0.134, *p(FDR)* = .027) and 17 networks (*r* = -0.135, *p(FDR)* = .027). No other correlations were found. These findings confirm the robustness of the negative relationship between PC2 and DMN_μV_, even when using the Schaefer atlas.

*4. Controlling for head movement and DMN size in correlation analyses*

    To account for potential confounding factors in the relationship between DMN state transitions and resilience scores, we included both mean framewise displacement (FD_mean_) and ROI size (DMN_size_) as covariates. This adjustment was necessary because DMN_rMSSD_ showed a positive correlation with head movement (FD_mean_) and a negative correlation with ROI size (DMN_size_). Despite controlling for these variables, DMN_rMSSD_ maintained significant negative correlations with resilience measures, specifically CD-RISC (*r* = -0.180, *p(FDR)* = .002), BRS (*r* = -0.150, *p(FDR)* = .008), and PC1 (*r* = -0.160, *p(FDR)* = .005).

*5. Additional analyses of Salience Networks from Schaefer atlas and Resilience*

To further evaluate the specificity of DMN-resilience association, we analyzed mean velocity within saliency networks defined in the Schaefer 100-parcel atlas. Both Salience/Ventral Attention A (SalVenAttnA: *r(334)* = -0.131, *p(FDR)* <.05) and Salience/Ventral Attention B (SalVenAttnB: *r(334)* = -0.194, *p(FDR)* <.001) showed significant negative correlation with PC2 (Fig. S5). These findings suggest that individuals with higher self-perceived resilience may show greater regulation of salience-driven reactivity, potentially maintaining more stable internal states even in the face of salient or distracting external cues.

To better understand the relationship between salience and DMN network findings, we also examined the spatial overlap between the salience network parcels from the Schaefer atlas and the DMN regions defined by the AAL3 atlas used in our primary analyses. We found that 16.52% of voxels in SalVenAttnA and 15.37% in SalVenAttnB overlapped with the AAL3-defined DMN regions. This moderate overlap raises the possibility that the observed correlations between salience network transitional rates and PC2 may, in part, be driven by shared regions with the DMN, further underscoring the need for future work to disentangle inter-network contributions to psychological resilience. To further probe this anatomical and functional overlap, we conducted exploratory follow-up analyses. For SalVenAttnA, when isolating the non-DMN ROIs, the correlation with PC2 became non-significant (*r(334)* = -0.042, *p(FDR)* =.566), whereas the DMN-overlapping portion retained a similar effect size and significant (*r(334)* = -0.123, *p(FDR)* <.05). This suggests that the SalVenAttnA-PC2 association was primarily driven by its DMN-overlapping voxels. In contrast, for SalVenAttnB, the non-DMN portion still showed a significant, though attenuated, correlation (*r(334)* = -0.136, *p(FDR)* <.05), while the DMN-overlapping part did not (*r(334)* = -0.084, *p(FDR)* =.205), indicating that the observed SalVenAttnB-PC2 association may reflect the point contribution of both DMN and non-DMN components. These findings collectively support the specificity of DMN involvement while also acknowledge meaningful functional interaction with salience-related regions.

Together with the negative association observed between the sensory network and PC2 in the main analysis, this convergence suggests that lower transition rates in externally oriented systems including perceptual and salience-related networks, may also relate to an individual’s self-perceived resilience, potentially by supporting more stable integration of external input during internally focused states.

**SM References**

[Borg, I., & Groenen, P. (2003). Modern multidimensional scaling: theory and applications. *Journal of Educational Measurement*, *40*(3), 277–280.](https://sciwheel.com/work/bibliography/6736572)

[Schaefer, A., Kong, R., Gordon, E. M., Laumann, T. O., Zuo, X.-N., Holmes, A. J., Eickhoff, S. B., et al. (2018). Local-Global Parcellation of the Human Cerebral Cortex from Intrinsic Functional Connectivity MRI. *Cerebral Cortex*, *28*(9), 3095–3114.](https://sciwheel.com/work/bibliography/4939207)


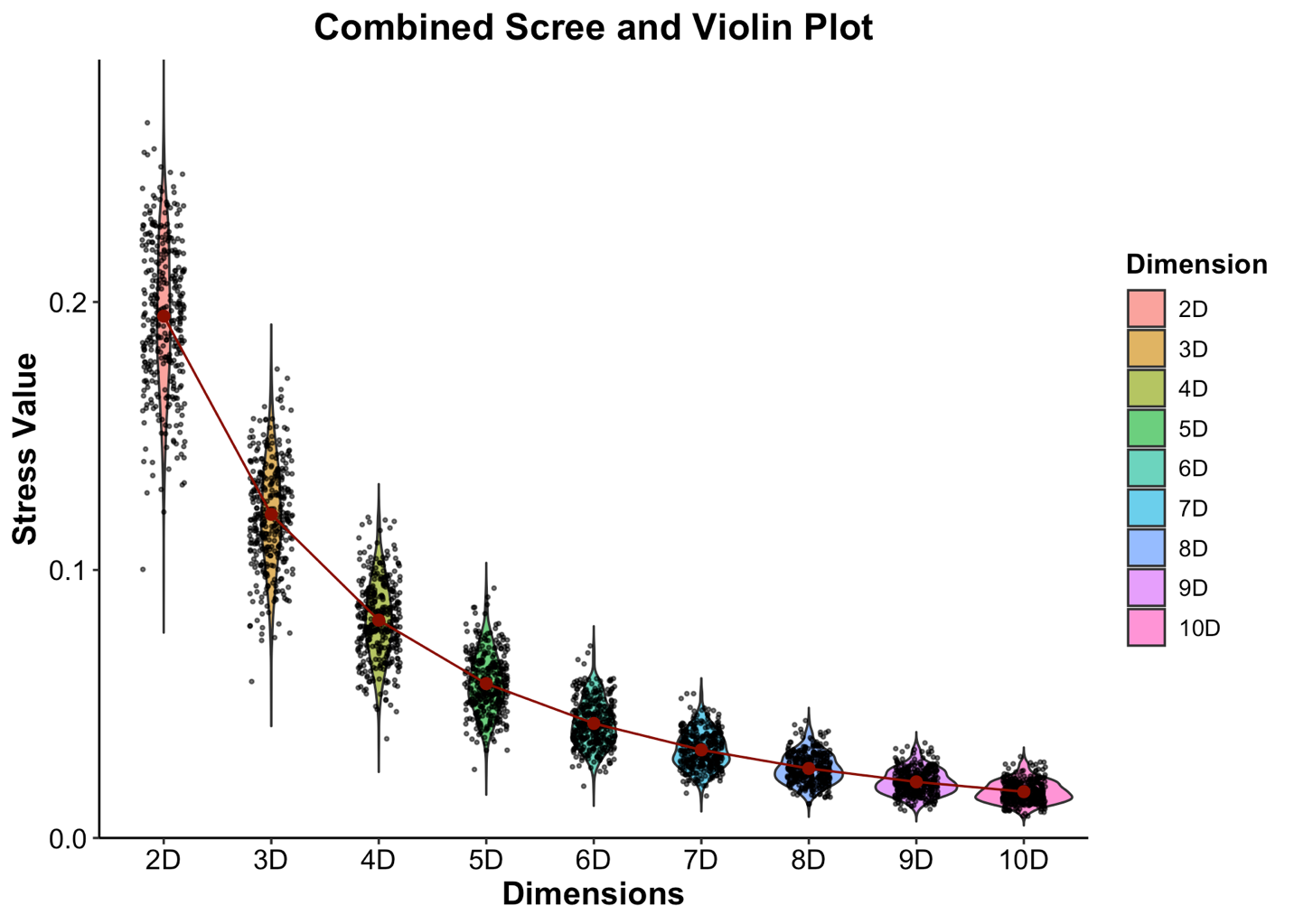


**Figure S1.** **Combined Scree and Violin Plot of MDS projection stress Values.** The black dots (individual values) and violin plots depict the distribution of stress values for different dimensional sizes, showing the variability at each dimensional size. Also, overlaid is the scree plot (red points and line) showing the average MDS projection stress values for each dimension, highlighting the trend across dimensional sizes.


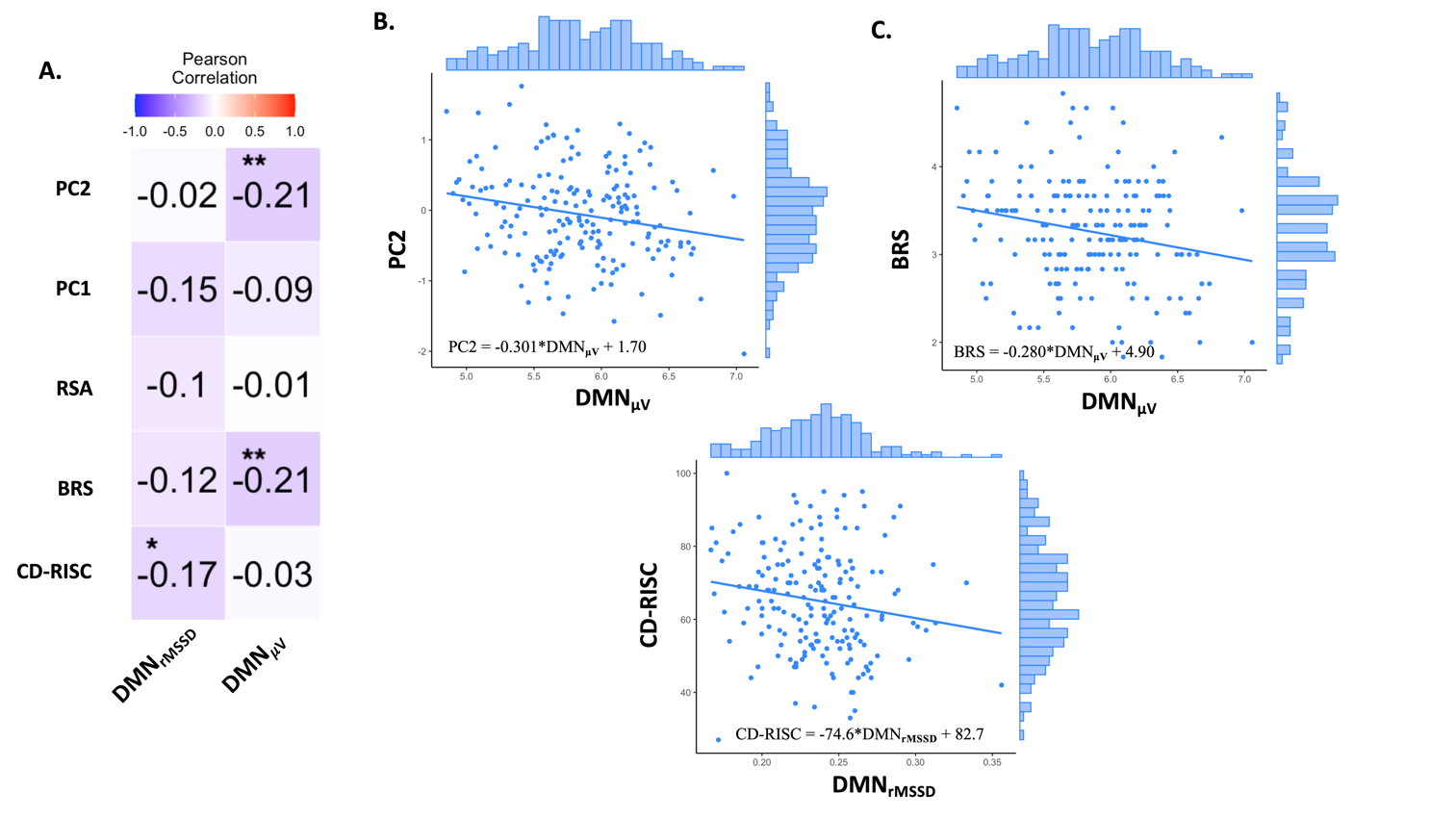


**Figure S2.** **Correlation analyses between DMN functional transition indices and psychological resilience measures for sub-batch 1 (N = 189). (A)** Heatmap for Pearson’s correlations between DMN functional transition indices (DMN_𝜇V_ and DMN_rMSSD_) and psychological resilience measures, and PCs 1 and 2 for sub-batch 1 (N = 189). **(B)** shows the scatterplots of significant correlations with distribution histograms on the axes. * denotes p(FDR) < 0.05. Equations denote regression functions of the effect of the DMN indices on the resilience measures (trend lines in plots). FDR: False Discovery Rate; CD-RISC: Connor-Davidson Resilience Scale; BRS: Brief Resilience Scale; RSA: Resilience Scale for Adults; PC1, PC2: Principal Components 1 and 2; DMN_𝜇V_: Mean default-mode network multi-voxel state transition velocity; DMN_rMSSD_: Root mean square of successive differences in default-mode network (time series averaged across voxels).


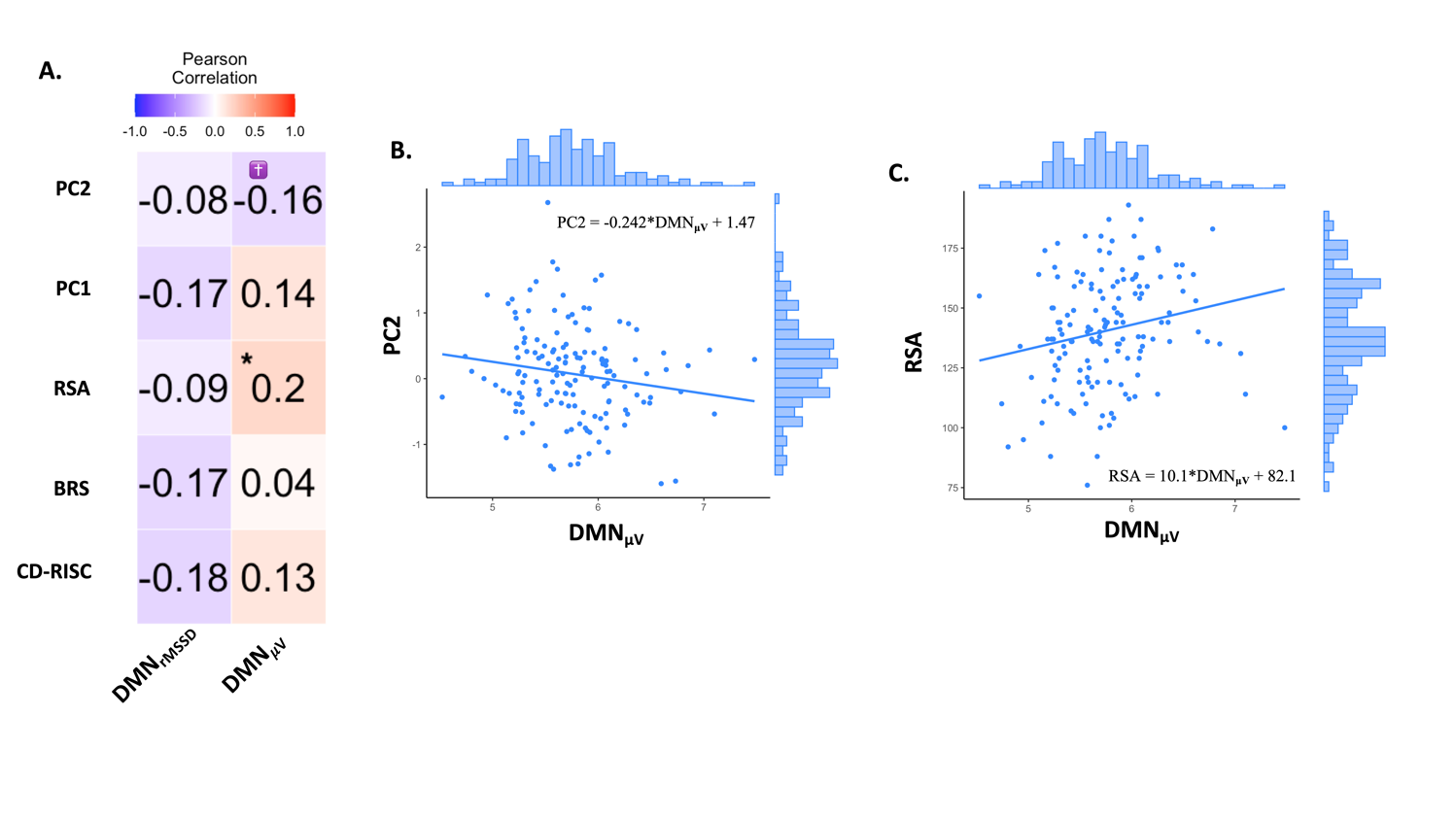


**Figure S3. Correlation analyses between DMN functional transition indices and psychological resilience measures for sub-batch 2 (N = 147). (A)** Heatmap for Pearson’s correlations between DMN functional transition indices (DMN_𝜇V_ and DMN_rMSSD_) and psychological resilience measures, and PCs 1 and 2 for sub-batch 2 (N = 147). **(B)** shows the scatterplots of the correlation between DMN_𝜇V_ and PC2 with distribution histograms on the axes. **(C)** to **(F)** show the scatterplots of significant correlations. * denotes p(FDR) < 0.05, and † denotes p(uncorrected) = 0.066. Equations denote regression functions of the effect of the DMN indices on the resilience measures (trend lines in plots). FDR: False Discovery Rate; CD-RISC: Connor-Davidson Resilience Scale; BRS: Brief Resilience Scale; RSA: Resilience Scale for Adults; PC1, PC2: Principal Components 1 and 2; DMN_𝜇V_: Mean default-mode network multi-voxel state transition velocity; DMN_rMSSD_: Root mean square of successive differences in default-mode network (time series averaged across voxels).

**
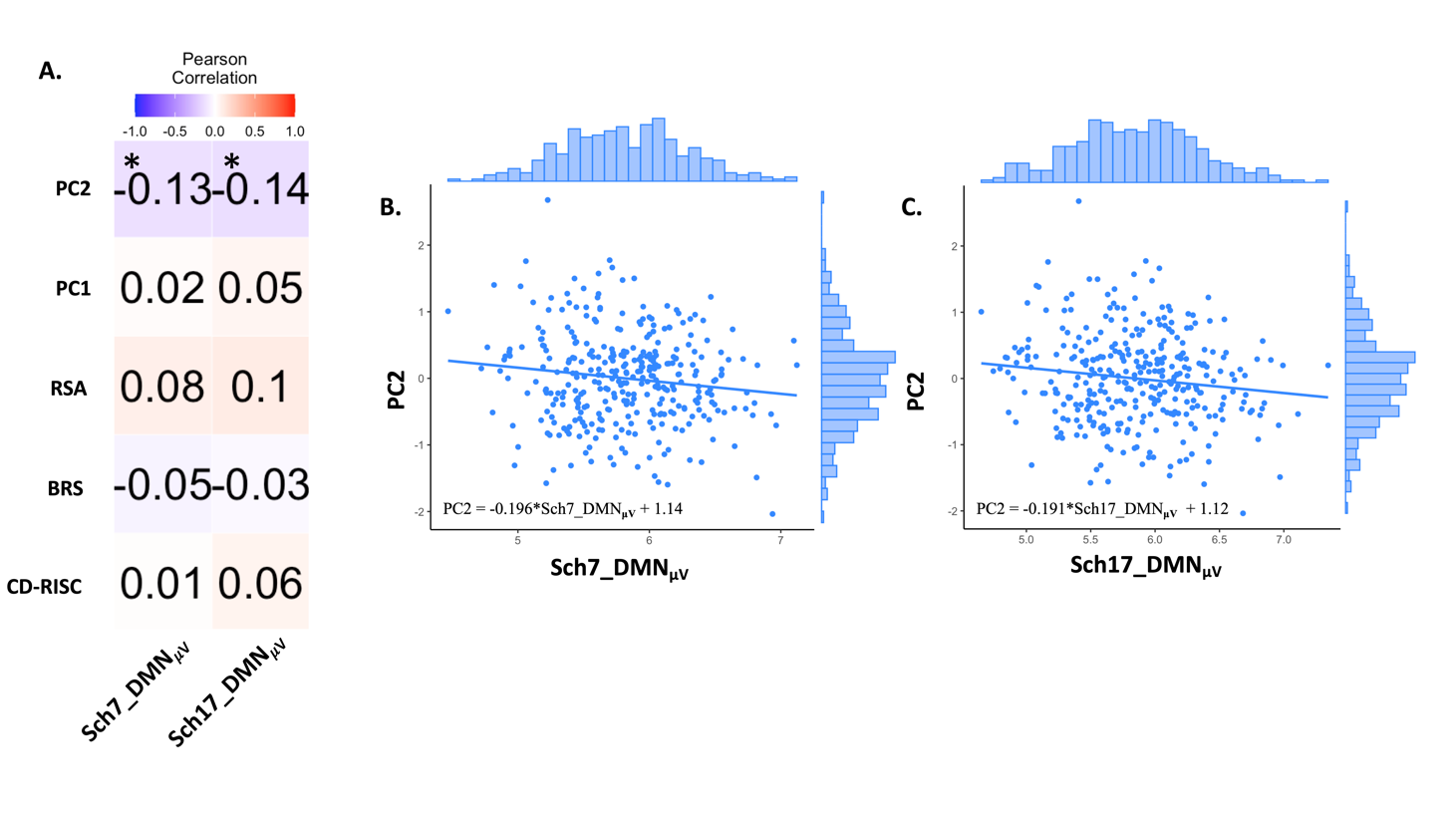
**

**Figure S4**. **Correlation analyses between DMN functional transition indices and psychological resilience measures using Schaefer 7 networks and 17 networks for the entire dataset (N = 336). (A)** Heatmap for Pearson’s correlations between DMN functional transition indices (Sch7_DMN_𝜇V_ and Sch17_DMN_𝜇V_) and psychological resilience measures, and PCs 1 and 2. **(B)** and **(C)** show the scatterplots of significant correlations with distribution histograms on the axes. * denote p(FDR) < 0.05. Equations denote regression functions of the effect of the DMN indices on the resilience measures (trend lines in plots). FDR: False Discovery Rate; CD-RISC: Connor-Davidson Resilience Scale; BRS: Brief Resilience Scale; RSA: Resilience Scale for Adults; PC1, PC2: Principal Components 1 and 2 (see Table 1); DMN_𝜇V_: Mean default-mode network multi-voxel state transition velocity.


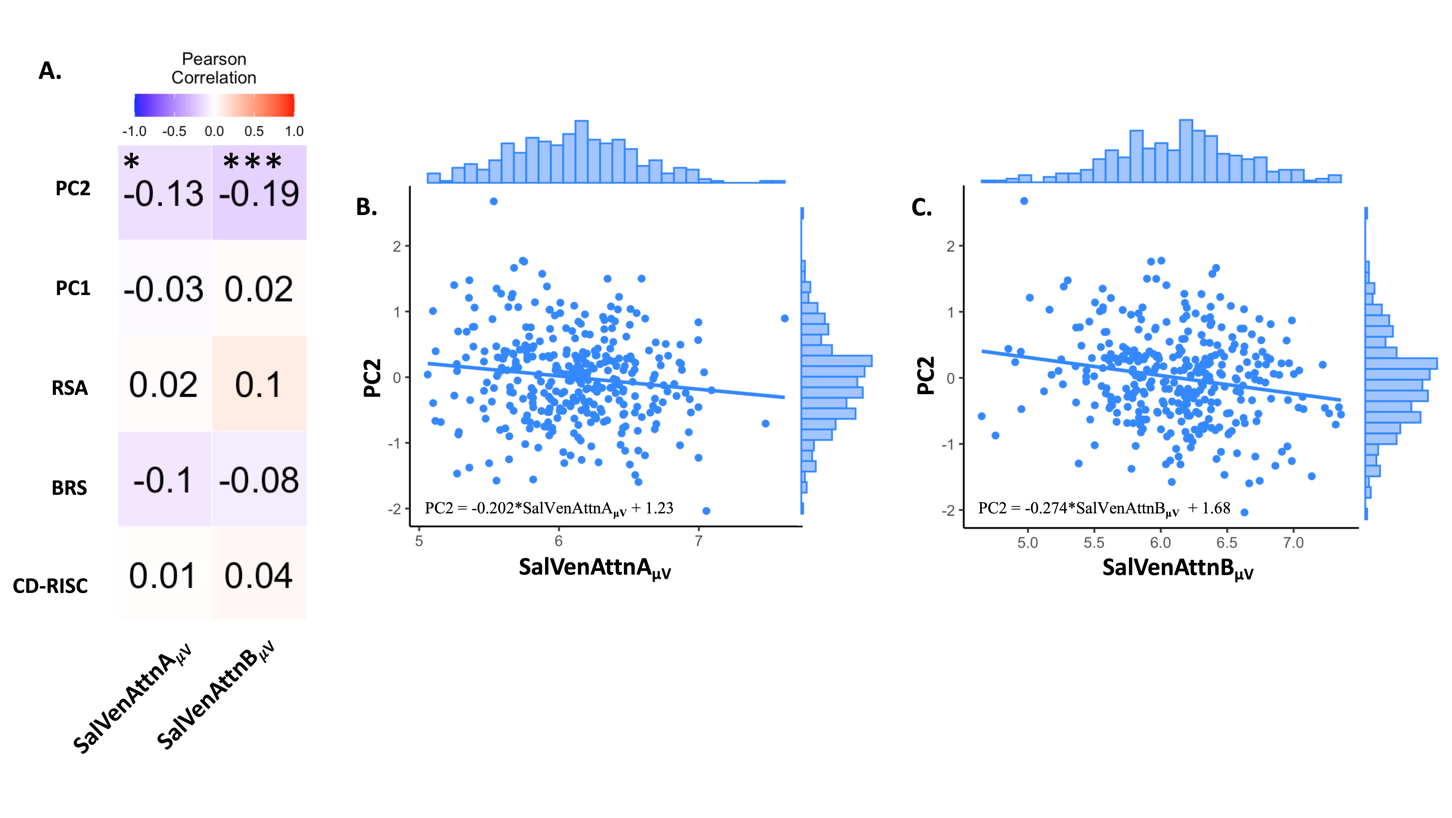


**Figure S5**. **Correlation analyses between salience network functional transition indices and psychological resilience measures using Schaefer 17 networks for the entire dataset (N = 336). (A)** Heatmap for Pearson’s correlations between Salience Ventral Attention A (SalVenAttnA _𝜇V_) and Salience Ventral Attention B (SalVenAttnB _𝜇V_) functional transition indices, and psychological resilience measures, and PCs 1 and 2. **(B)** and **(C)** show the scatterplots of significant correlations with distribution histograms on the axes. *, *** denote p(FDR) < 0.05, 0.001, respectively. Equations denote regression functions of the effect of the DMN indices on the resilience measures (trend lines in plots). FDR: False Discovery Rate; CD-RISC: Connor-Davidson Resilience Scale; BRS: Brief Resilience Scale; RSA: Resilience Scale for Adults; PC1, PC2: Principal Components 1 and 2 (see Table 1); SalVenAttnA _𝜇V_: Mean Salience Ventral Attention A network multi-voxel state transition velocity; SalVenAttnB _𝜇V_: Mean Salience Ventral Attention B network multi-voxel state transition velocity.

**Table S1.** Automated anatomical labeling version 3 (AAL3) anatomical regions with classifications into default mode network (DMN), and control sensory and action network regions-of-interest used in this present study.

| Primary network | Secondary network | Regions | AAL3 label |
| --- | --- | --- | --- |
| Sensory network |  |  |  |
|  | Primary sensory areas |  |  |
|  |  | Heschl’s Gyrus | 83,84 |
|  |  | Superior temporal gyrus | 85,86 |
|  |  | Calcarine gyrus | 47,48 |
|  |  | Olfactory gyrus | 17,18 |
|  |  | Postcentral gyrus | 61,62 |
|  | Secondary sensory areas |  |  |
|  |  | Middle temporal gyrus | 89,90 |
|  |  | Inferior occipital gyrus | 57,58 |
|  |  | Middle occipital gyrus | 55,56 |
|  |  | Superior occipital gyrus | 53,54 |
|  |  | Cuneus | 49,50 |
|  |  | Lingual Gyrus | 51,52 |
|  |  | Inferior parietal lobule | 65,66 |
|  |  | Paracentral lobule | 73,74 |
|  | Association areas |  |  |
|  |  | Inferior temporal gyrus | 93,94 |
|  |  | Fusiform gyrus | 59,60 |
|  |  | Superior parietal lobule | 63,64 |
|  | Medial temporal system |  |  |
|  |  | Parahippocampal gyrus | 43,44 |
|  |  | Hippocampus | 41,42 |
|  | Limbic system |  |  |
|  |  | Amygdala | 45,46 |
|  |  | Superior temporal pole | 87,88 |
|  |  | Middle temporal pole | 91,92 |
|  |  | Rolandic Operculum | 13,14 |
| Default mode network |  |  |  |
|  | Default mode network |  |  |
|  |  | Angular gyrus | 68,70 |
|  |  | Supramarginal gyrus | 67,68 |
|  |  | Precuneus | 71,72 |
|  |  | Posterior cingulate | 39,40 |
|  |  | Superior medial frontal | 19,20 |
|  |  | Medial orbitofrontal | 21,22,25,26 |
|  |  | Rectus gyrus | 23,24 |
| Action network |  |  |  |
|  | Monitoring system |  |  |
|  |  | Anterior cingulate gyrus | 35,36,151~156 |
|  |  | Insula | 33,34 |
|  | Executive system |  |  |
|  |  | Superior frontal gyrus | 3,4 |
|  |  | Middle frontal gyrus | 5,6 |
|  |  | Inferior frontal gyrus | 7~12 |
|  |  | Striatal | 75~78 |
|  |  | Middle cingulate gyrus | 37,38 |
|  |  | Lateral orbitofrontal | 27~32 |
|  | Premotor areas |  |  |
|  |  | Supplementary motor area | 15,16 |
|  | Primary motor areas |  |  |
|  |  | Precentral gyrus | 1,2 |

**Table S2.** Factor loadings of the PCs from principal component analysis of the five RSA subscales and other two resilience measures (CD-RISC and BRS) with percentages of total variance accounted for in parentheses and the means of the measures.

|  | Mean (SD) | PC1_2_  (54.6%) | PC2_2_  (14.5%) | PC3_2_  (11.0%) |
| --- | --- | --- | --- | --- |
| RSA-Family cohesion | 34.7 (7.18) | 0.504 | 0.456 | 0.709 |
| RSA-Social competence | 19.4 (5.16) | 0.662 | 0.450 | -0.397 |
| RSA-Social resources | 43.5 (7.36) | 0.668 | 0.539 | -0.189 |
| RSA-Personal Strength | 27.6 (6.45) | 0.879 | -0.270 | -0.042 |
| RSA-Future structured style | 18.6 (5.30) | 0.798 | -0.228 | -0.120 |
| CD-RISC | 63.3 (14.4) | 0.868 | -0.248 | -0.007 |
| BRS | 3.24 (0.66) | 0.723 | -0.351 | 0.237 |
| SD: Standard Deviation; PC: Principal Components; CD-RISC: Connor-Davidson Resilience Scale; BRS: Brief Resilience Scale; RSA: Resilience Scale for Adults. | | | | |
